# Quantitative evaluation of parotid gland dysfunction in patients with hyposalivation using magnetic resonance imaging mapping technique: Quantification of Parotid gland function using MRI

**DOI:** 10.1186/s12903-025-05873-y

**Published:** 2025-04-11

**Authors:** Chena Lee, Ari Lee, Yoon Joo Choi, Sang-Sun Han, Hee Woo Cho, Kug Jin Jeon

**Affiliations:** 1 Department of Oral and Maxillofacial Radiology, Yonsei University College of Dentistry, 50-1 Yonsei-ro, 03722 Seoul, Republic of Korea; 2 Department of Preventive Medicine, Yonsei University College of Medicine, 50-1 Yonsei-ro, 03722 Seoul, Republic of Korea; 3 Oral Science Research Center, Yonsei University College of Dentistry, 50-1 Yonsei-ro, 03722 Seoul, Republic of Korea; 4 Department of Radiology, Yongin Severance Hospital, Yonsei University College of Medicine, 363 Dongbaekjukjeon-daero, Gyeonggi-do 16995 Yongin-si, Republic of Korea

**Keywords:** Multi-dynamic multi-echo sequence, Magnetic resonance imaging, Hyposalivation, Salivary gland, Relaxation map, Dry mouth

## Abstract

**Objective:**

This study aimed to assess the feasibility of quantitative magnetic resonance imaging (MRI) employing the multi-dynamic multi-echo (MDME) technique as a diagnostic modality for evaluating glandular dysfunction in patients with hyposalivation.

**Methods:**

The MDME technique generated T1, T2, and proton density (PD) maps of the parotid gland, allowing for the simultaneous acquisition of values from the respective mappings. The Mann–Whitney U-test was used to compare the hyposalivation and control groups, and receiver operating characteristic (ROC) curve analysis was performed.

**Results:**

A total of 71 patients who underwent MDME MRI were reviewed and categorized into hyposalivation patients (*n* = 32) and healthy controls (*n* = 25). The average T1, T2 and PD value of the gland in the hyposalivation group were 606.92 ms, 91.85 ms, and 82.52 pu, respectively, whereas those in the control group were 628.08 ms, 80.69 ms, and 91.12 pu, respectively. The T2 and PD values were significantly different between the hyposalivation and control groups. The cut-off T2 value was 85.75 ms (AUC = 0.8131, *p* < 0.0001) and the cut-off PD value was 81.55 pu (AUC = 0.7588, *p* = 0.0009).

**Conclusions:**

T2 and PD values derived from the MDME technique demonstrated strong potential for detecting parotid gland dysfunction in hyposalivation patients. These findings suggest that MDME-based quantitative MRI mapping shows promise in evaluating hyposalivation of the parotid gland and could become a valuable diagnostic tool in clinical settings.

**Supplementary Information:**

The online version contains supplementary material available at 10.1186/s12903-025-05873-y.

## Introduction

Saliva is essential for oral and systemic health, consisting mainly of water (∼ 99%) along with electrolytes, proteins, enzymes, and antimicrobial agents. It facilitates lubrication, digestion, pH buffering, antimicrobial defense, and enamel remineralization. Salivary dysfunctions primarily include hyposalivation and xerostomia [1, 2]. Hyposalivation is an objectively measured decrease in saliva production, often caused by medications, systemic diseases (e.g., Sjögren’s syndrome), radiation therapy, or aging [2]. Xerostomia, in contrast, is the subjective sensation of dry mouth, which can occur even with normal salivary flow. Management includes saliva substitutes, sialagogues, hydration, and behavioral interventions [2]. A clear understanding of saliva and its dysfunctions is essential for effective diagnosis and treatment.

Currently, hyposalivation can be diagnosed in clinics using the passive drool test [3]. During the test, saliva is collected from the patient for a certain period by stimulating its flow using gum or candy. Hyposalivation can be diagnosed when the unstimulated salivary flow rate is ≤ 0.1 mL/min or the stimulated salivary flow rate is ≤ 0.5–0.7 mL/min [1]. This passive saliva drool test is currently the gold standard for detecting a reduction in salivary flow [4]. Löfgren et al. emphasized the importance of functional assessment and the need for accurate diagnostic tools for hyposalivation to effectively identify patients requiring treatment for dryness [5].

Salivary gland scintigraphy using technetium-99 m pertechnetate is a clinically widely used functional imaging modality for assessing gland, however, it dose not show anatomical information. As magentic resonance imagings (MRI) offers an advantage in providing functional information collaborated with anatomical details, researchers are exploring optimal MRI sequences for evaluating salivary gland [6–11]. Diffusion-weighted imaging (DWI) has frequently been studied for the quantitative evaluation of salivary gland function. DWI is a technique that measures the movement of water molecules within tissues. When water molecules diffuse actively, the signal is lower, resulting in darker images, while restricted water diffusion leads to brighter images. These variations in diffusion can be quantified and presented as apparent diffusion coefficient (ADC) values through mathematical formulas. According to previous studies, ADC values have been found to correlate significantly with the level of salivary flow within the salivary glands, making DWI a valuable tool for assessing salivary gland function, particularly in patients with gland dysfunction [6, 7, 9–12]. However, due to the low resoluation of DWI, location of precise region of the gland may not possible [13]. Another approach uses fat fraction measurements in the salivary gland to evaluate its function, as the gland undergoes fatty degeneration when its function declines. However, studies highlight the limitation of this technique, as the fat fraction in the gland can vary according to factors such as age and body mass index, even in non-dysfunctional glands [8, 14]. Overall, an additional imaging technique is required to present a strong correlation with the functional aspects of the gland [8].

Quantification of MRI parameters, including T1, T2, and proton density (PD), provides tissue-specific values that reflect the intrinsic characteristics of tissue properties under a magnetic field. These values have been used in the diagnosis of various diseases, such as liver cirrhosis and Alzheimer’s disease [15–17]. Additionally, as demonstrated by Zhou et al. [18] and Vidmar et al. [19], T2 mapping has shown potential in assessing parotid gland function following radiotherapy for head and neck cancers, suggesting its applicability to salivary glands. However, the application of these techniques to salivary gland evaluation has been limited due to the lengthy acquisition times required for traditional mapping methods. Moreover, acquiring T1 and PD maps is even more challenging than T2 mapping because of the significantly longer imaging times, which has hindered their research and application. The recent development of the multi-dynamic multi-echo (MDME) sequence has addressed these limitations, enabling the simultaneous acquisition of T1, T2, and PD maps in approximately half the time required by conventional methods. This method generates various contrast images by manipulating multiple scanning parameters during a single acquisition [20].

Therefore, This study aimed to assess the feasibility of quantitative MRI employing the MDME technique as a diagnostic modality for evaluating glandular dysfunction in patients with hyposalivation.

## Materials and methods

### Selection of participants

This study utilized a retrospective design with a convenience sample, including patients who underwent MRI in dental hospital between July 2020 and December 2022, and only images obtained using the MDME method were included. Patients with orofacial pain conditions, such as temporomandibular joint disease, trigeminal neuralgia, or Sjögren’s syndrome, underwent imaging examinations following the hospital’s imaging protocol. The clinical records of patients were thoroughly reviewed. Patients who underwent an initial assessment for subjective oral dryness through a questionnaire were included. Patients who reported symptoms of dryness and had a whole saliva flow rate of less than 0.7 mL/min were classified into the hyposalivation group. Those who did not report dryness were categorized as the control group [1, 5]. Patients with salivary gland disorders, including tumors, those taking medications that affect gland function, or those who had undergone head and neck radiotherapy were excluded. MRI with artifacts or incomplete coverage of the gland structure were excluded.

### Ethics approval and consent to participate

This study was conducted in accordance with the ethical standards outlined in the Declaration of Helsinki and relevant national regulations. The study protocol was reviewed and approved by the Institutional Review Board (IRB) of Yonsei University Dental Hospital (Approval Number: 2-2021-0058). Given the retrospective nature of the study and the use of anonymized data, the IRB waived the requirement for obtaining informed consent to participate. No identifiable patient data were included in the analysis or presentation of this study.

### Image acquisition and analysis

The MRI scans were performed using a 3.0-T scanner (Pioneer; GE Healthcare, Waukesha, WI, USA) with a large Flex coil. T1 and T2 relaxation times and PD values were obtained using the multivariate empirical mode decomposition method in the axial orientation, with the following imaging parameters for synthetic reconstruction: repetition time (TR), 4000 ms; echo time (TE), 21.3 and 85.2 ms; four different inversion times (TIs), 211, 611, 1811, and 3811 ms; field of view (FOV), 210 × 210 mm; acquisition matrix, 300 × 200; reconstructed voxel size, 0.7 × 1.0 mm; slice thickness, 2.5 mm; and echo train length, 12. The image acquisition time was 6 min and 40 s.

**Fig. 1:**
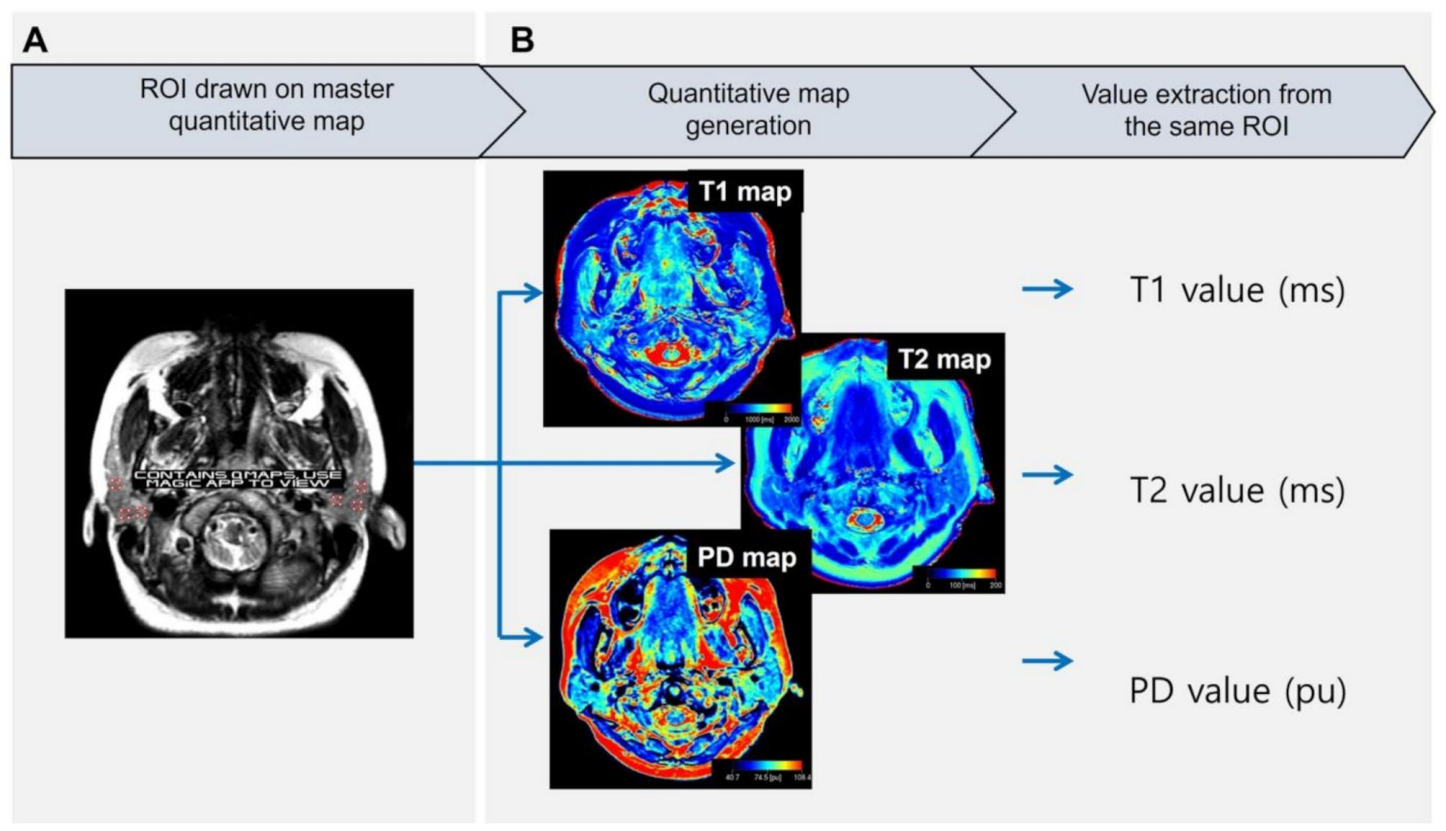
(**A**) Schematic view of the region-of-interest (ROI) selection on a quantitative map using the multi-dynamic multi-echo technique. ROIs of 0.1 cm^2^ with a square shape were established on the master quantitative map. (**B**) The T1 and T2 and proton density (PD) values were extracted simultaneously from each ROI and the average value was used for analysis. (ms, miliseconds; pu, percentage units)

The overall image analysis was conducted by two oral and maxillofacial radiologists, each with over 10 years of experience. Measurement calibration was performed using two samples before the measurements, and during the experiment, they discussed each step until a consensus was reached. The axial slice displaying the largest area of both parotid glands was selected, following the methodology of previous studies [8, 21]. To include parenchymal tissue without intra-parotid lymph nodes or vessels, square-shaped regions-of-interest (ROI) with a size of 0.1 cm^2^ were placed on the deep, middle, and superficial parenchyma of the gland on the master quantitative map (Fig. 1A). The console then displayed the same ROI on the T1, T2, and PD maps. For each map, the corresponding T1, T2, and PD values were extracted from the defined ROI (Fig. 1B). These values were obtained by measuring the T1 and T2 relaxation times in milliseconds (ms) and PD value in percentage units (pu) within the selected region. The values from the six ROIs of each individual map (T1, T2, and PD) were then averaged for further analysis, based on a previous study that found no significant difference between the right and left parotid glands [8, 14, 22].

### Statistical analysis

The T1, T2, and PD values of the hyposalivation group were compared with those of the control group. Shapiro-Wilk normality tests were performed, and since the normality assumption was not satisfied, the Mann–Whitney U test was used for comparison, with a confidence interval of 95%. A *p*-value of < 0.05 was considered statistically significant. Receiver operating characteristic (ROC) analysis was conducted for variables that showed significant differences between the two groups. Diagnostic cut-off values were determined using Youden’s index. Comparisons between Area under the curve (AUC) was conducted using the method suggested by Hanley and McNeil [23]. Statistical analyses were performed using GraphPad Prism version 9.5.1 for Windows (GraphPad Software, San Diego, CA, USA).

## Results

**Fig. 2:**
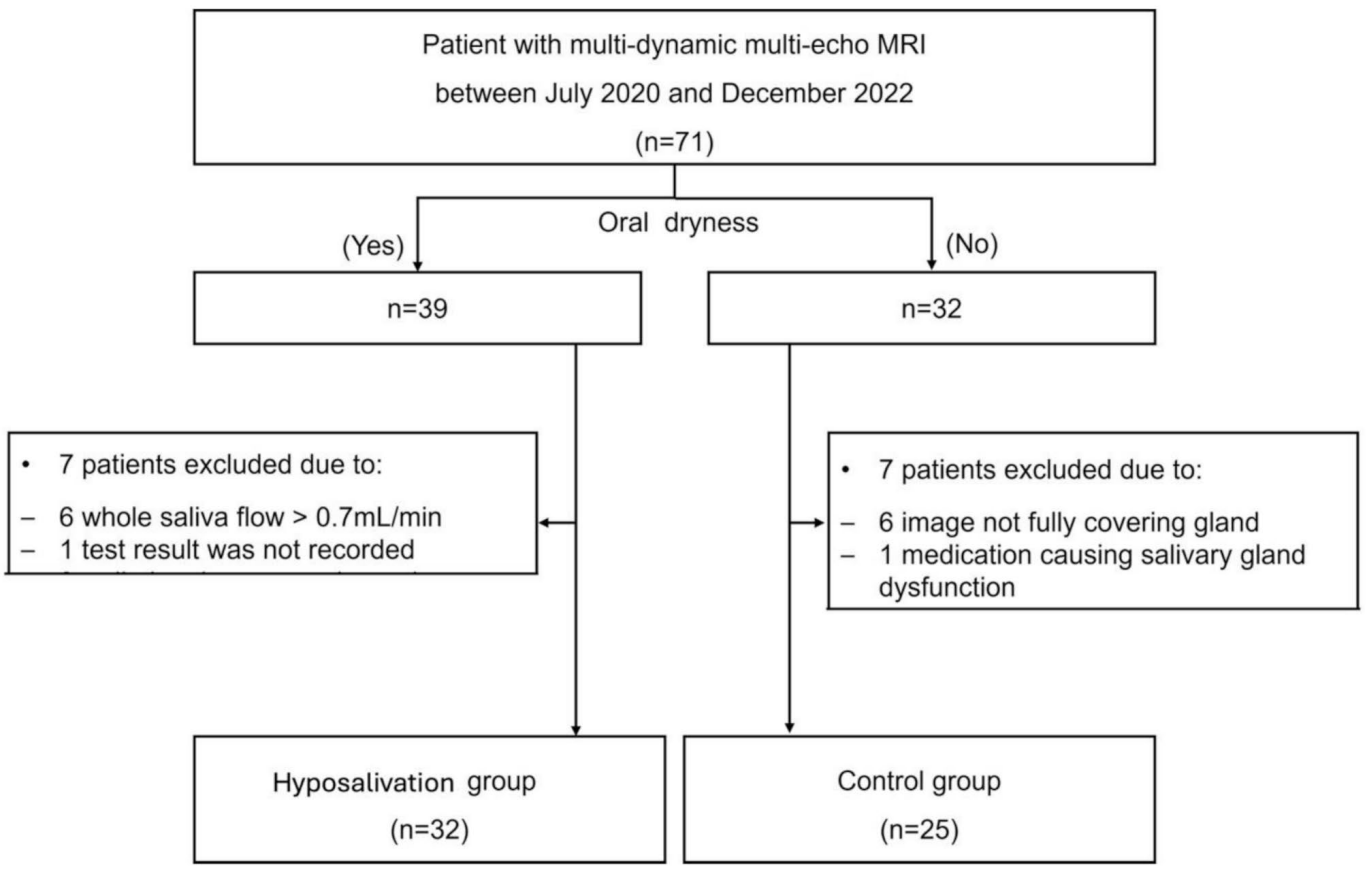
Flowchart showing patient selection and screening process. MRI, magnetic resonance imaging

Thirty-two patients with hyposalivation and 25 controls were included in this study (Fig. 2). The average age of patients included in the study was 49.39 ± 19.16 years with a male to female ratio of 1:3.75 (male, 12; female, 45). The patient information of each group, hyposalivation and control was described in Table 1.

**Table 1:** Patient information of overall study subjects

		Hyposalivation	Control
Age (Mean ± SD)		60.93 ± 10.48	34.60 ± 17.57
Sex	Male (*n*)	4	8
	Female (*n*)	28	17

Abbreviation: SD, standard deviation

**Fig. 3:**
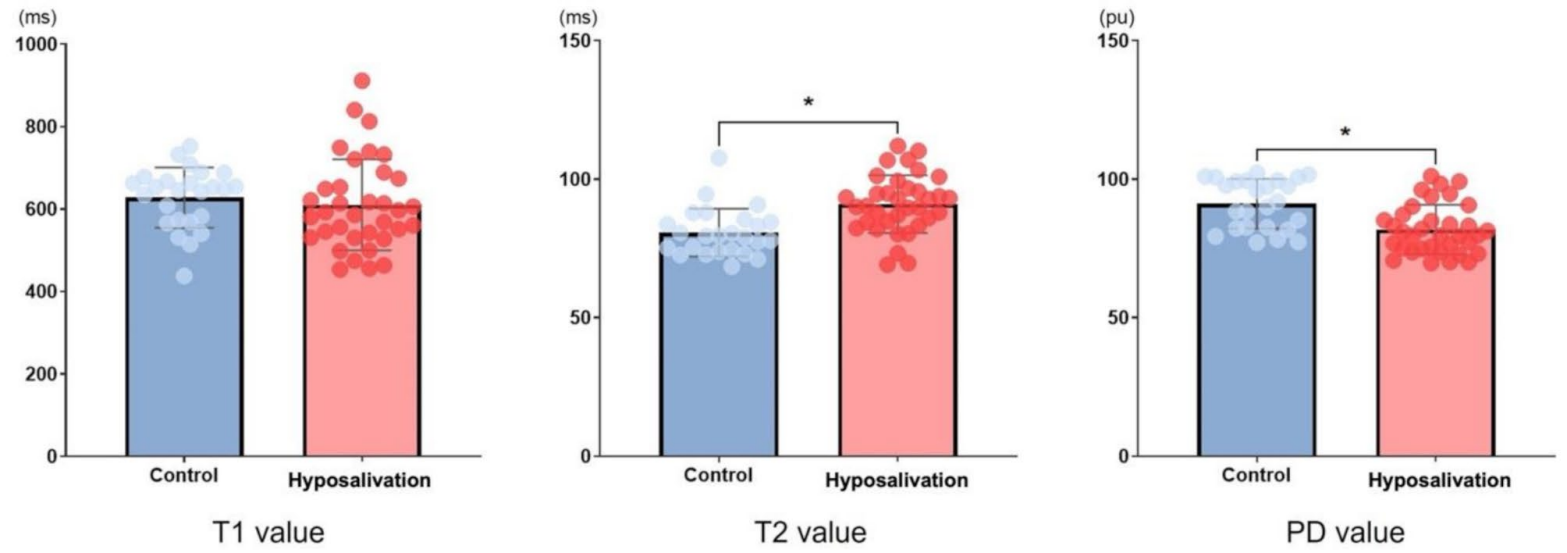
Comparison between patients with hyposalivation and control participants with respect to the T1 and T2 and proton density (PD) values. *Mann–Whitney U-test, 95% confidence interval. (ms, miliseconds; pu, percentage units)

**Fig. 4:**
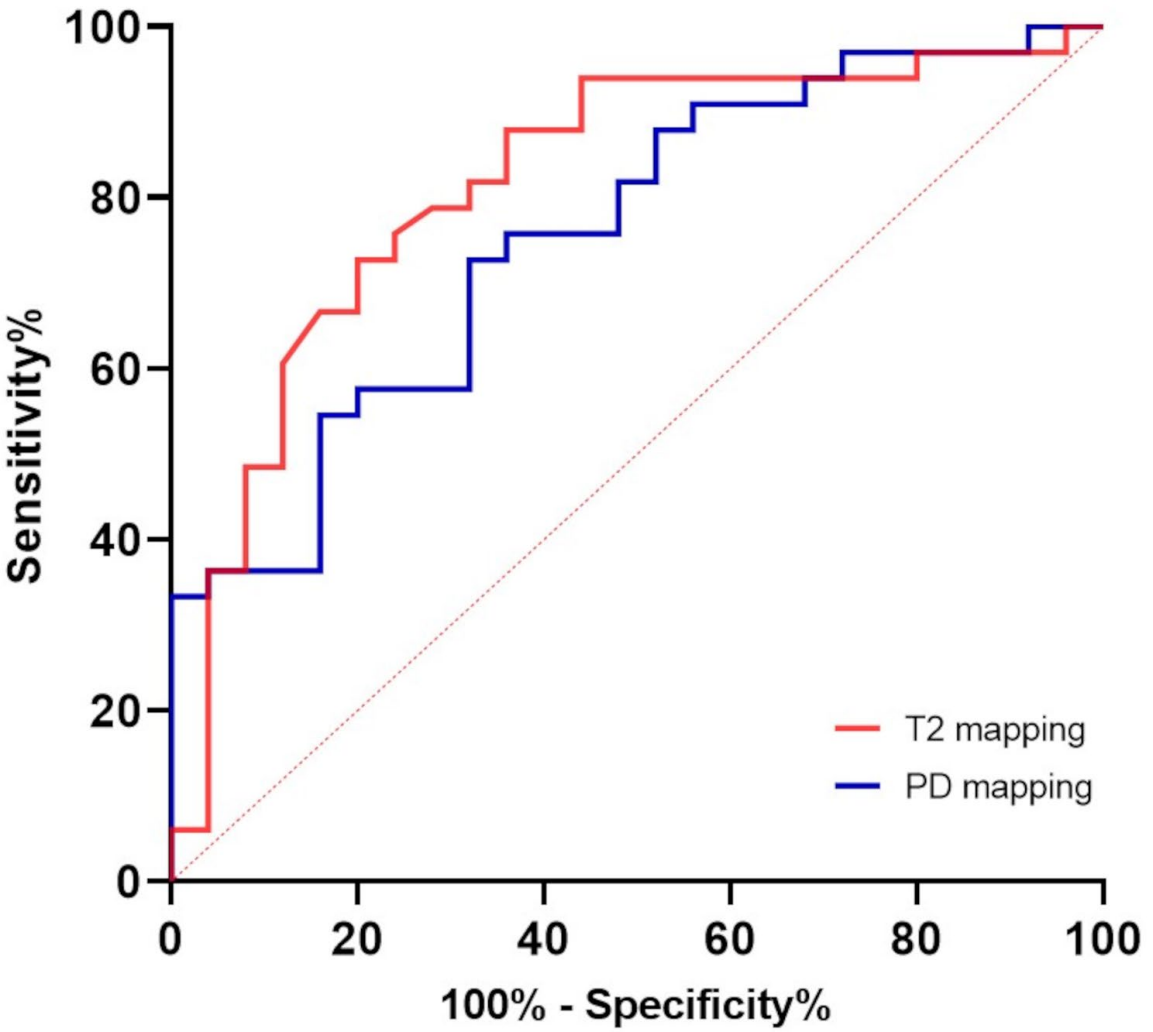
Receiver operating characteristic curve of T2 and proton density (PD) values for diagnosis of hyposalivation gland

The average T1, T2 and PD value of the gland in hyposalivation group were 606.92 ms, 91.85 ms, and 82.52 pu, respectively. The average T1, T2 and PD value of the control group were 628.08 ms, 80.69 ms, and 91.12 pu, respectively (Fig. 3). The T2 and PD values were significantly different between the hyposalivation and control groups, whereas there was no significant difference in T1 value between the two groups. The cut-off value was 85.75 ms (AUC = 0.8131, *p* < 0.0001) for T2 map and 81.55 pu (AUC = 0.7588, *p* = 0.0009) for PD map (Fig. 4). Number of true positive, true negative, false positive, false negative, sensitivity, specificity, and AUC of T2 and PD value were described in Table 2. The PD map demonstrated higher specificity, but lower sensitivity compared to the T2 map. The diagnostic performances of T2 and PD maps were similar (*p* = 0.5301).

**Table 2:** Comparitive analysis of the diagnostic performance of T2 and PD values

	True positive (*n*)	True negative (*n*)	False postive (*n*)	False negative (*n*)	Cut-off value	Sensitivity (%)	Specificity (%)	Area under the curve	Standard error	95% confidence interval	*p*-value^¶^
T2 value	24	20	5	8	85.75 ms	71.88	80.00	0.8131	0.0593	0.6968–0.9295	0.5301
PD value	17	21	4	15	81.55 pu	56.25	84.00	0.7588	0.0629	0.6355–0.8820	

Abbreviation: PD, proton density

^¶^Comparisons between area under the curve (AUC) was conducted using the method suggested by Hanley and McNeil [23]

## Discussion

This study demonstrated that quantitative MRI mapping, particularly the MDME technique, has potential as a diagnostic tool for evaluating parotid gland dysfunction in patients with hyposalivation. Specifically, T2 and PD values showed significant differences between hyposalivation patients and healthy controls, with T2 demonstrating “good” diagnostic performance and PD showing “fair” performance [24]. Both metrics were more effective for ruling out hyposalivation, given their higher specificity compared to sensitivity.

Quantitative MRI parameters, such as T1 and T2 relaxation time values, reflect intrinsic tissue characteristics and have been extensively studied for their biological significance in various organs. In this study, the T2 map emerged as a particularly useful diagnostic model for assessing gland function in hyposalivation patients. Another study on radiation-induced hyposalivation using T2 mapping showed that T2 values significantly increased continuously from pretreatment to mid- and post-radiation therapy [18]. Also, the previous literatures, T2 value have been used to diagnose malignant tumors, along with blood oxygen levels and iron quantification [25–27]. Studies suggested that functional impairment in the salivary glands with ongoing parenchymal degeneration and congestion manifests as a T2 value difference.

The PD value, which reflects free water contrast [15], was also shown to be lower in dysfunctional glands, aligning with the understanding of impaired salivary gland function. Meanwhile, T1 values did not show a significant difference between patients and healthy controls. Previous research suggested that T1 values may vary depending on the stage of tissue fibrosis or dysfunction [28]. For example, T1 increased in acute disease stages and decreased in chronic conditions [29]. In this study, the wide range of T1 values in hyposalivation patients may be attributed to the inclusion of individuals with varying stages of gland dysfunction, without strict disease stage control.

In terms of diagnostic potential, both T2 and PD maps were more effective for ruling out hyposalivation due to their higher specificity compared to sensitivity. Notably, while the T2 map demonstrated higher overall diagnostic accuracy, the PD map exhibited greater specificity than the T2 map. This suggested that the PD map may be particularly useful in correctly identifying individuals without the condition, thereby reducing false positives. Moreover, this imaging approach showed the potential to play a crucial role in distinguishing between xerostomia, a subjective symptom of dry mouth, and hyposalivation, which reflects an actual glandular functional impairment. Since gland dysfunction can manifest in varying degrees, the ability to differentiate between these conditions is clinically significant. By providing objective measurements of gland function, these imaging techniques could enhance diagnostic precision and improve patient management in cases where subjective symptoms alone may not accurately reflect glandular health.

Other studies using MRI techniques like DWI and fat fraction quantification provided mixed results for evaluating salivary gland function [6, 8, 14, 30, 31]. While DWI can reflect functional changes, its clinical accuracy could be limited due to challenges in identifying reliable ROIs, especially in the parotid gland, which is prone to air artifacts [7, 30, 32, 33]. By contrast, the MDME method used in this study provided higher resolution MRI maps, enabling more accurate ROI placement and better diagnostic performance [34].

Fat fraction quantification has also been explored for evaluating salivary gland dysfunction [8, 14, 31, 35]. Some studies reported good diagnostic performance, with AUC values similar to our findings [36]. However, measuring the fat fraction would be considered a more indirect approach, as it reflects the degenerative changes in the tissue caused by gland dysfunction rather than directly assessing the dysfunction itself [8, 14, 35]. Therefore, fat fraction diganostic techqniue presetned some limitation especially for patient’s weight or age factor might significantly affect gland fat fraction [31, 33].

This study is the first to apply the MDME technique to the diagnosis of hyposalivation, demonstrating its potential as a promising tool for assessing gland function through T1, T2, and PD maps. The technique offers a non-invasive alternative to conventional diagnostic methods, enabling targeted evaluation of specific salivary glands and reducing patient burden compared to salivary flow rate tests. Additionally, it provides a less invasive option than procedures like salivary gland biopsy.

However, the study had several limitations. The relatively small sample size from a single institution restricted the generalizability of the findings. Due to the small sample size and the retrospective nature of the study, it was not possible to analyze hyposalivation based on its underlying causes. Additionally, the normal and hyposalivation groups were not analyzed with equal sample sizes or matched for age and sex distribution. Recent previous research reported that the MDME method is reliable, which supports the credibility of our study [20]. However, while prior study was conducted using phantom, our study applied the technique to actual patients, highlighting a key difference. Therefore, future multicenter studies comparing imaging data obtained from different MRI systems are required to provide stronger evidence for the clinical reliability of this technique. In addition, further prospective studies comparing flow rate measurements of individual salivary glands with corresponding imaging values could enhance the reliability and accuracy of the diagnostic approach, further validating the technique’s clinical utility.

In conclusion, T2 relaxation time and PD values derived from the MDME technique demonstrated strong potential for detecting parotid gland dysfunction in hyposalivation patients. These findings suggest that MDME-based quantitative MRI mapping could become a valuable diagnostic tool in clinical settings. Further studies with larger and more diverse populations are warranted to validate and refine these results.

## Electronic supplementary material

Below is the link to the electronic supplementary material.

Supplementary Material 1

## Data Availability

All data used in this study are considered patient information and may be provided upon request, subject to approval through contact with the institution’s IRB coordinator (dentalirb@yuhs.ac).

## Abbreviations

MRI: Magnetic resonance imaging
MDME: Multi-dynamic multi-echo
DWI: Diffusion-weighted imaging
ADC: Apparent diffusion coefficient
PD: Proton density
ROI: Regions-of-interest
TR: Repetition time
TE: Echo time
AUC: Area under the curve

## References

[1] Villa A, Connell CL, Abati S. Diagnosis and management of Xerostomia and hyposalivation. Ther Clin Risk Manag. 2014;11:45–51. 25653532 10.2147/TCRM.S76282 PMC4278738

[2] Hummelsheim MZ, Hamacher S, Hagemeier A, et al. Care need and dry mouth as risk indicators for impaired taste and smell. Sci Rep. 2021;11:20419. 34650210 10.1038/s41598-021-99978-3 PMC8516854

[3] Navazesh M, Kumar SK. Measuring salivary flow: challenges and opportunities. J Am Assoc. 2008;139:S35–40. 10.14219/jada.archive.2008.0353 18460678

[4] Fallon BS, Chase TJ, Cooke EM, et al. The use of BokaFlo™ instrument to measure salivary flow. BMC Oral Health. 2021;21:1–7. 33845818 10.1186/s12903-021-01477-4 PMC8042968

[5] Löfgren CD, Wickström C, Sonesson M et al. A systematic review of methods to diagnose oral dryness and salivary gland function. BMC Oral Health. 2012;12:1-16.6. 10.1186/1472-6831-12-29 PMC3573918 22870895

[6] Dirix P, De Keyzer F, Vandecaveye V, et al. Diffusion-weighted magnetic resonance imaging to evaluate major salivary gland function before and after radiotherapy. Int J Radiat Oncol Biol Phys. 2008;71:1365–71. 18355977 10.1016/j.ijrobp.2007.12.011

[7] Kato H, Kanematsu M, Toida M, et al. Salivary gland function evaluated by diffusion-weighted MR imaging with gustatory stimulation: preliminary results. J Magn Reson Imaging. 2011;34:904–9. 21837780 10.1002/jmri.22729

[8] Lee A, Choi YJ, Jeon KJ, et al. Impact of physiological parameters on the Parotid gland fat fraction in a normal population. Sci Rep. 2023;13:990. 36653427 10.1038/s41598-023-28193-z PMC9849206

[9] Juan CJ, Chang HC, Hsueh CJ, et al. Salivary glands: echo-planar versus PROPELLER Diffusion-weighted MR imaging for assessment of ADCs. Radiology. 2009;253:144–52. 19789257 10.1148/radiol.2531082228

[10] Sumi M, Takagi Y, Uetani M, et al. Diffusion-weighted echoplanar MR imaging of the salivary glands. Am J Roentgenol. 2002;178:959–65. 11906883 10.2214/ajr.178.4.1780959

[11] Yoshino N, Yamada I, Ohbayashi N, et al. Salivary glands and lesions: evaluation of apparent diffusion coefficients with split-echo diffusion-weighted MR imaging—initial results. Radiology. 2001;221:837–42. 11719687 10.1148/radiol.2213010131

[12] Munhoz L, Ramos EADA, Im DC, Hisatomi M, Yanagi Y, Asaumi J, Arita ES. Application of diffusion-weighted magnetic resonance imaging in the diagnosis of salivary gland diseases: a systematic review. Oral Surg Oral Med Oral Pathol Oral Radiol. 2019;128:280–310. 31029591 10.1016/j.oooo.2019.02.020

[13] Koh DM, Collins DJ. Diffusion-weighted MRI in the body: applications and challenges in oncology. AJR Am J Roentgenol. 2007;188(6):1622–35. 17515386 10.2214/AJR.06.1403

[14] Su GY, Wang CB, Hu H, et al. Effect of laterality, gender, age and body mass index on the fat fraction of salivary glands in healthy volunteers: assessed using iterative decomposition of water and fat with echo asymmetry and least-squares Estimation method. Dentomaxillofac Radiol. 2019;48:20180263. 30306806 10.1259/dmfr.20180263 PMC6476364

[15] Shah B, Anderson SW, Scalera J, et al. Quantitative MR imaging: physical principles and sequence design in abdominal imaging. Radiographics. 2011;31:867–80. 21571662 10.1148/rg.313105155

[16] Tang X, Cai F, Ding DX, et al. Magnetic resonance imaging relaxation time in Alzheimer’s disease. Brain Res Bull. 2018;140:176–89. 29738781 10.1016/j.brainresbull.2018.05.004

[17] Lee C, Choi YJ, Jeon KJ, et al. Synthetic magnetic resonance imaging for quantitative parameter evaluation of temporomandibular joint disorders. Dentomaxillofac Radiol. 2021;50:20200584. 33544630 10.1259/dmfr.20200584 PMC8231687

[18] Zhou N, Chu C, Dou X, Chen W, He J, Yan J, Zhou Z, Yang X. Early evaluation of radiation-induced Parotid damage in patients with nasopharyngeal carcinoma by T2 mapping and mDIXON quant imaging: initial findings. Radiat Oncol. 2018;13:22. 29422068 10.1186/s13014-018-0970-9 PMC5806279

[19] Vidmar J, Cankar K, Groselj M, Finderle Z, Sersa I. Assessment of hyperbaric oxygenation treatment response in Parotid glands by *T*_2_ mapping following radiotherapy for head and neck tumours. Radiol Oncol. 2022;11:56:60–8. 10.2478/raon-2022-0001 PMC8884852 35148472

[20] Zheng Z, Yang J, Zhang D, et al. The effect of scan parameters on T1, T2 relaxation times measured with multi-dynamic multi-echo sequence: a Phantom study. Phys Eng Sci Med. 2022;45:657–64. 35553390 10.1007/s13246-022-01128-0 PMC9239947

[21] Ito K, Muraoka H, Hirahara N, Sawada E, Tokunaga S, Kaneda T. Quantitative assessment of the Parotid gland using computed tomography texture analysis to detect Parotid sialadenitis. Oral Surg Oral Med Oral Pathol Oral Radiol. 2022;133:574–81. 34953759 10.1016/j.oooo.2021.10.022

[22] Patel RR, Carlos RC, Midia M, Mukherji SK. Apparent diffusion coefficient mapping of the normal Parotid gland and Parotid involvement in patients with systemic connective tissue disorders. AJNR Am J Neuroradiol. 2004;25:16–20. 14729521 PMC7974152

[23] Hanley JA, McNeil BJ. A method of comparing the areas under receiver operating characteristic curves derived from the same cases. Radiology. 1983;148:839–43. 6878708 10.1148/radiology.148.3.6878708

[24] Muller MP, Tomlinson G, Marrie TJ, et al. Can routine laboratory tests discriminate between severe acute respiratory syndrome and other causes of community-acquired pneumonia? Clin Infect Dis. 2005;40:1079–86. 15791504 10.1086/428577 PMC7107805

[25] Gomori J, Grossman R, Drott HJA. JoR. MR relaxation times and iron content of thalassemic spleens: an in vitro study. Am J Roentgenol. 1988;150:567–69. 3257611 10.2214/ajr.150.3.567

[26] Kaltwasser J, Gottschalk R, Schalk K, et al. Non-invasive quantitation of liver iron‐overload by magnetic resonance imaging. Br J Haematol. 1990;74:360–63. 2334643 10.1111/j.1365-2141.1990.tb02596.x

[27] Kolnagou A, Yazman D, Economides C, et al. Uses and limitations of serum ferritin, magnetic resonance imaging T2 and T2* in the diagnosis of iron overload and in the ferrikinetics of normalization of the iron stores in thalassemia using the international committee on chelation Deferiprone/deferoxamine combination protocol. Hemoglobin. 2009;33:312–22. 19814677 10.3109/03630260903213231

[28] Chen W, Su GY, Zhou Y, et al. Longitudinal multiparametric MRI assessment of irradiated salivary gland in a rat model: correlated with histological findings. J Magn Reson Imaging. 2021;54:1730–741. 34278649 10.1002/jmri.27836

[29] Chai JW, Lin YC, Chen JH, et al. In vivo magnetic resonance (MR) study of fatty liver: importance of intracellular ultrastructural alteration for MR tissue parameters change. J Magn Reson Imaging. 2001;14:35–41. 11436212 10.1002/jmri.1148

[30] Chu C, Feng Q, Zhang H, et al. Whole-volume ADC histogram analysis in Parotid glands to identify patients with Sjögren’s syndrome. Sci Rep. 2019;9:1–8. 31270382 10.1038/s41598-019-46054-6 PMC6610085

[31] Jeon KJ, Park Y, Jeong H, et al. Parotid gland evaluation of menopausal women with Xerostomia using the iterative decomposition of water and fat with echo asymmetry and least-squares Estimation (IDEAL-IQ) method of MRI: a pilot study. Dentomaxillofac Radiol. 2023;52:20220349. 36695352 10.1259/dmfr.20220349 PMC10170170

[32] Regier M, Ries T, Arndt C, et al. Sjögren’s syndrome of the Parotid gland: value of diffusion-weighted echo-planar MRI for diagnosis at an early stage based on MR sialography grading in comparison with healthy volunteers. RöFo. 2009;181:242–48. 19229790 10.1055/s-0028-1109105

[33] Xu X, Su G, Hu H, et al. Effects of regions of interest methods on apparent coefficient measurement of the Parotid gland in early Sjögren’s syndrome at 3T MRI. Acta Radiol. 2017;58:27–33. 26987670 10.1177/0284185116637245

[34] Zou M, Zhou Q, Li R, Hu M, Qian L, Yang Z, Zhao J. Image quality using synthetic brain MRI: an age-stratified study. Acta Radiol. 2023;64:2010–23. 36775871 10.1177/02841851231152098

[35] Kise Y, Chikui T, Yamashita Y, et al. Clinical usefulness of the mDIXON quant the method for Estimation of the salivary gland fat fraction: comparison with MR spectroscopy. Br J Radiol. 2017;90:20160704. 28707990 10.1259/bjr.20160704 PMC5858799

[36] van Dijk LV, Thor M, Steenbakkers RJ, et al. Parotid gland fat related magnetic resonance image biomarkers improve prediction of late radiation-induced Xerostomia. Radiother Oncol. 2018;128:459–66. 29958772 10.1016/j.radonc.2018.06.012 PMC6625348

